# Uncover a microbiota signature of upper respiratory tract in patients with SARS-CoV-2 + 

**DOI:** 10.1038/s41598-023-43040-x

**Published:** 2023-10-06

**Authors:** Massimo Bellato, Marco Cappellato, Francesca Longhin, Claudia Del Vecchio, Giuseppina Brancaccio, Anna Maria Cattelan, Paola Brun, Claudio Salaris, Ignazio Castagliuolo, Barbara Di Camillo

**Affiliations:** 1 Department of Information Engineering, University of Padova, 35131 Padova, Italy; 2 Department of Molecular Medicine, University of Padova, 35121 Padova, Italy; 3 Infectious Diseases Unit, University Hospital Padova, 35128 Padova, Italy; 4 Microbiology and Virology Unit, University Hospital Padova, 35121 Padova, Italy; 5 Department of Comparative Biomedicine and Food Science, University of Padova, 35020 Legnaro (PD), Italy

**Keywords:** Network topology, Metagenomics, Prognostic markers

## Abstract

The outbreak of Coronavirus disease 2019 (COVID-19), caused by SARS-CoV-2, forced us to face a pandemic with unprecedented social, economic, and public health consequences. Several nations have launched campaigns to immunize millions of people using various vaccines to prevent infections. Meanwhile, therapeutic approaches and discoveries continuously arise; however, identifying infected patients that are going to experience the more severe outcomes of COVID-19 is still a major need, to focus therapeutic efforts, reducing hospitalization and mitigating drug adverse effects. Microbial communities colonizing the respiratory tract exert significant effects on host immune responses, influencing the susceptibility to infectious agents. Through 16S rDNAseq we characterized the upper airways’ microbiota of 192 subjects with nasopharyngeal swab positive for SARS-CoV-2. Patients were divided into groups based on the presence of symptoms, pneumonia severity, and need for oxygen therapy or intubation. Indeed, unlike most of the literature, our study focuses on identifying microbial signatures predictive of disease progression rather than on the probability of infection itself, for which a consensus is lacking. Diversity, differential abundance, and network analysis at different taxonomic levels were synergistically adopted, in a robust bioinformatic pipeline, highlighting novel possible taxa correlated with patients’ disease progression to intubation.

## Introduction

The severe acute respiratory syndrome coronavirus 2 (SARS-CoV-2) caused a worldwide extraordinary public health threat infecting millions of people. A striking trait of SARS-CoV-2 infection is the wide variability of clinical manifestations in infected people. Thus, infections fluctuate from asymptomatic cases or minimal self-limiting illness to severe pneumonia and death. Although many factors seem to correlate with infection severity, such as age, gender, body mass index, the presence of comorbidities, genetic and immune system function, the factors determining infection outcome are still not well understood^1^. The upper respiratory tract is the portal of entry of SARS-CoV-2 infection that eventually can reach the lung parenchyma causing the most serious clinical manifestations. Infection of mucosal surfaces occurs in the presence of its endogenous microbiota, and the bidirectional interplay between host, microbiota, and pathogen contributes to infection success and pathogenesis. It is widely accepted that knowing in depth the different aspects of the relationship between microbiota and disease prognosis leads to great advantages in terms of preventive and therapeutic medicine^2,3^.

The literature related to respiratory tract microbiota and COVID-19 is relatively discordant and a consensus is still far to be achieved. Many studies report no significant associations between infected patients and healthy controls^4–6^, nor consider clusters divided by pathology severity^7^. On the other hand, for example, Shilts et al.^8^ observe a clear trend in alpha and beta diversity between healthy controls and patients who develop severe disease (although not statistically significant). Conversely, in Saha et al.^9^ the beta diversity is significantly different between positive and negative subjects, whereas Prasad et al.^10^ find that alpha diversity is significant between infected subjects and the control group, but not between symptomatic and asymptomatic subjects. Finally, Mostafa et al.^11^ found alpha and beta diversity estimates that were significantly different between infected subjects and the healthy control group.

Overall, despite a large number of studies, only a few consistent associations between the nasopharyngeal microbiome and COVID-19 severity, symptoms, or outcome are present in the vast COVID-19 literature^12^. The contradictory results might steam from different analysis methods used since, as demonstrated in Calgaro et al.^13^ and Nearing et al.^14^, differential abundance (DA) methods can produce different results.

In this work, we analyze the nasopharyngeal tract microbiota of 194 subjects infected by SARS-CoV-2 focusing on infection severity and analyzing the data both in terms of microbial diversity and differential abundance (DA)^15^. Additionally, we corroborate the analysis with a network inference analysis, a novel strategy, here applied for the first time to microbiome nasopharyngeal sequencing data of SARS-CoV-2 positive patients, that could clarify which are the significant interactions driving the signature of severe outcomes.

Our dataset consists of 16S rDNA-seq obtained from 192 nasopharyngeal swabs from subjects positive for the first time for SARS-CoV-2 search. The main objective of the project is to search for an association between the SARS-CoV-2 virus infection and the taxonomic composition of the patients’ nasopharyngeal microbiota, with a specific focus on disease progression biomarkers. To achieve this goal, the 16S rRNA gene was sequenced and analyzed to determine the possible associations with patients’ metadata. In this way it could be determined whether the presence of a certain taxa contributes to the infection severity outcome or, on the contrary, prevents it.

It is worth noting that metadata were updated during the disease, but the samples were analyzed and sequenced immediately after the detection of SARS-CoV-2. Therefore, only the relationship between the microbiota detected at the time of the first control swab and the virus infection was taken into consideration. Any dysbiosis caused by hospitalization or therapies is not monitored in this dataset.

## Results

For statistical analysis, patients were grouped based on gender, age, and severity of infection (no symptom, upper respiratory tract infection but no pneumonia, moderate infection with lung involvement, severe pneumonia). While the main outcome is related to the presence of symptoms of the infection, further analyses were also carried out considering three different levels of pneumonia and the need for oxygenation. The main characteristics of the patients involved in the study are summarized in Table 1.

**Table 1:** Study cohort composition. Patient numerosity for each covariate under study at enrollment.

Covariate	Levels	Gender	#	Age				
				20–39	40–59	60–79	80–99	Mean ± SD
Main outcome	Asymptomatic: 36	M F	22 14	4 6	8 3	7 3	3 2	56 ± 20 50 ± 22
	Symptomatic:156	M F	105 51	20 9	42 15	29 17	14 10	56 ± 18 60 ± 19
	Total: 192	M F	127 65	24 15	50 18	36 20	17 12	56 ± 18 58 ± 20
Pneumonia	Mild: 89	M F	65 24	15 5	33 7	12 7	5 5	52 ± 17 59 ± 21
	Moderate: 50	M F	25 25	4 4	6 8	8 9	7 4	63 ± 18 60 ± 18
	Severe: 17	M F	15 2	1 0	3 0	9 1	2 1	64 ± 17 80 ± 14
	Total: 156	M F	105 51	20 9	42 15	29 17	14 10	56 ± 18 60 ± 19
Supplemental O_2_	Low/High-flow O_2_: 72 (intubated: 18)	M F	42 (16) 30 (2)	3 (1) 4 (0)	11 (4) 8 (1)	20 (9) 11 (1)	8 (1) 7 (0)	64 ± 16 (62 ± 17) 63 ± 18 (58 ± 24)

### Differences in whole bacterial composition

Alpha and Beta diversity analysis^16^ were carried out on three taxonomic levels, namely: amplicon sequence variant (ASV), genus, and species. Several metrics at different taxonomic resolutions were calculated to assess the overall microbial community diversity from various points of view.

Considering the intra-group mean species diversity (i.e., Alpha-diversity), Pielou’s and Richness (also known as Observed Features) metrics were compared and are reported in Table 2; at ASV taxonomy level, it was also possible to adopt the Faith phylogenetic-related metric, leveraging on the phylogenetic tree computed as described in “Materials and methods” section.

**Table 2:** Kruskal–Wallis *p* values on alpha-diversity metrics at different taxonomic resolution. Covariates are represented as reported in the “Materials and methods” section (“Data retrieval” section, “Patients’ metadata” paragraph): Gender (female F or male M); Outcome (symptomatic S or asymptomatic A); Pneumonia (mild m, moderate M or severe S); Supplemental O_2_ and Intubation (yes Y or no N).

	ASV			Species		Genus	
	*Faith*	*Pielou*	*Richness*	*Pielou*	*Richness*	*Pielou*	*Richness*
Gender (F/M)	**0.0007**	**0.030**	**0.0011**	**0.0125**	**0.0007**	**0.005**	**0.0005**
Outcome (S/A)	0.349	0.450	0.686	0.248	0.471	0.452	0.402
Pneumonia							
Mild versus moderate (m/M)	0.583	0.470	0.690	0.449	0.713	0.213	0.708
Mild versus severe (m/S)	0.817	0.741	0.566	0.785	0.612	0.940	0.659
Moderate versus severe (M/S)	0.452	0.966	0.408	0.889	0.323	0.578	0.347
All	0.895	0.445	0.862	0.512	0.854	0.398	0.880
Suppl.O_2_ (Y/N)	0.914	0.989	0.873	0.890	0.836	0.756	0.876
Intubation (Y/N)	0.647	0.865	0.537	0.414	0.425	0.642	0.427

Statistically significant *p* values (< 0.05) are highlighted in bold.

As reported in Table 2, Alpha diversity at ASV level showed significant p-values for the gender covariate through all the considered metrics (*p* < 0.05). However, this cannot be considered a disease-relevant finding, being rather related to behavioral (e.g., personal hygiene, smoking) or hormonal aspects.

**Figure 1:**
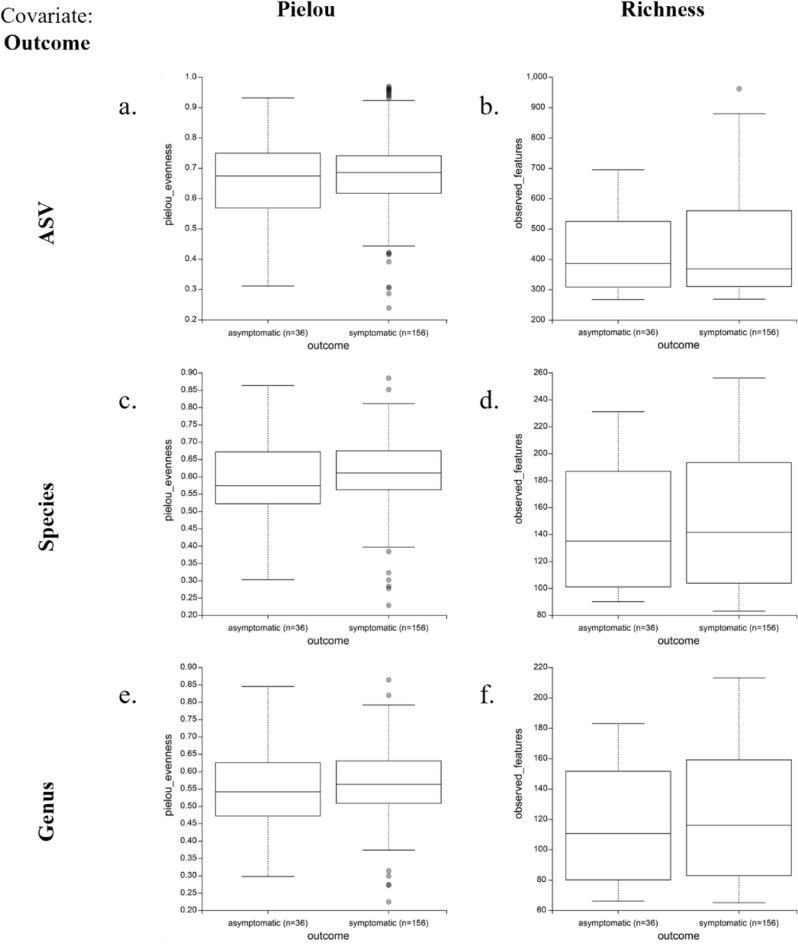
Alpha diversity analysis. Pielou’s and Richness metric at ASV, species and genus resolutions for Outcome (i.e., presence of symptoms) covariate.

Groups of patients characterized by the presence/absence of symptomatology had similar alpha diversity; the same pattern was also confirmed for pneumonia severity (either considering the three possible outcomes or dichotomizing severity in two classes) and endured therapy. This association was confirmed at both species and genus levels. As an example, evenness and richness distributions for the primary outcome, respectively provided by the Pielou and Observed Features metrics, are reported in Fig. 1. For plots related to the other covariates, see the Zenodo repository reported in the Data availability section.

**Figure 2:**
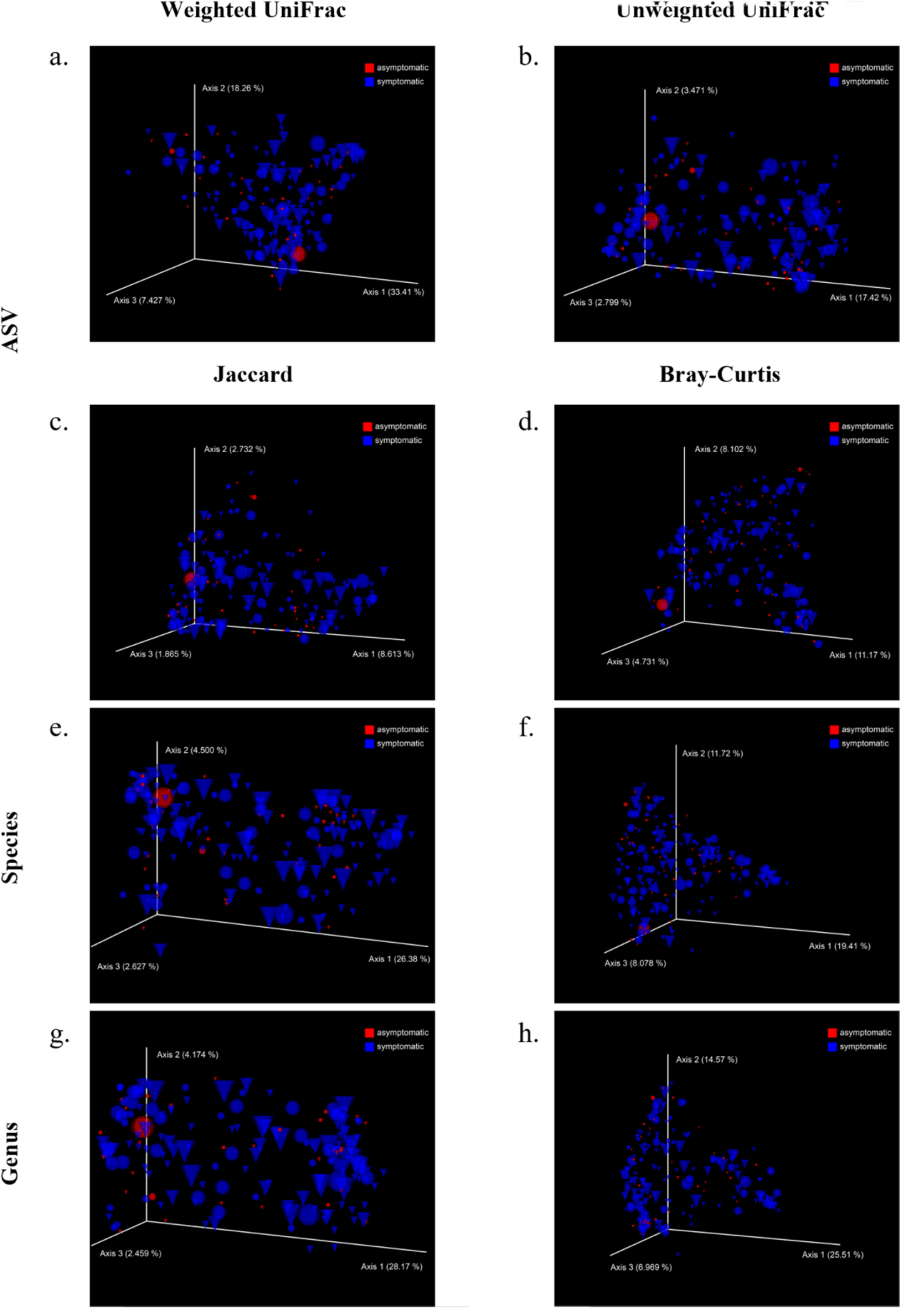
Beta diversity analysis. Emperor plots with axis computed as PCoA; gender is represented as spheres for females and cones for males; main outcome is represented as blue for symptomatic and red for asymptomatic; size correspond to increasing level of pneumonia severity (none, mild, moderate, severe).

The Beta diversity analysis was performed on the same groups of subjects at all the taxonomic resolution levels. The Bray–Curtis and Jaccard metrics were adopted, and Emperor plots were used to visualize sample profiles exploiting the Principal coordinates analysis (PCoA) as a dimensionality reduction technique. Additionally, weighted, and unweighted UniFrac distances were used at ASV taxonomic level, again leveraging on the knowledge of the phylogenetic tree (see “Bioinformatics pipeline” in the “Materials and methods” section). This analysis did not show significant results for any covariate, metric, or taxonomic resolution. Almost every metric showed a PCoA plot with clusters and accumulation spots, but none of them was clearly distinguishable through the covariates considered. Samples are distributed randomly in the three-dimensional space, without forming any cluster, as reported in Fig. 2. Therefore, we can conclude that accordingly to Beta diversity, SARS-CoV-2 infection does not affect the overall between-samples microbial community.

Taken together these results demonstrate that, at diagnosis, there are no hints, in terms of overall bacterial composition, about the future development of the disease. Although no global differences in bacterial diversity within the sample have been detected, this does not exclude the existence of differential abundance of individual taxa in different groups.

### Associated bacterial identification

Differential abundance (DA) analysis^15^ can potentially identify taxa that characterize patient’s microbiota and are associated with different symptom development. DA was carried out using MaAsLin2^17^. Results show that the genus *Ornithinimicrobium* is statistically significantly more abundant for patients undergoing intubation or that develop severe symptoms of pneumonia. Moreover, species *Ornithinimicrobium pekingense*, *Jonquetella anthropi* and a not classified species of the genus *Enterococcus* are statistically significantly more abundant, at species level, in patients that need intubation, as reported in Table 3.

**Table 3:**
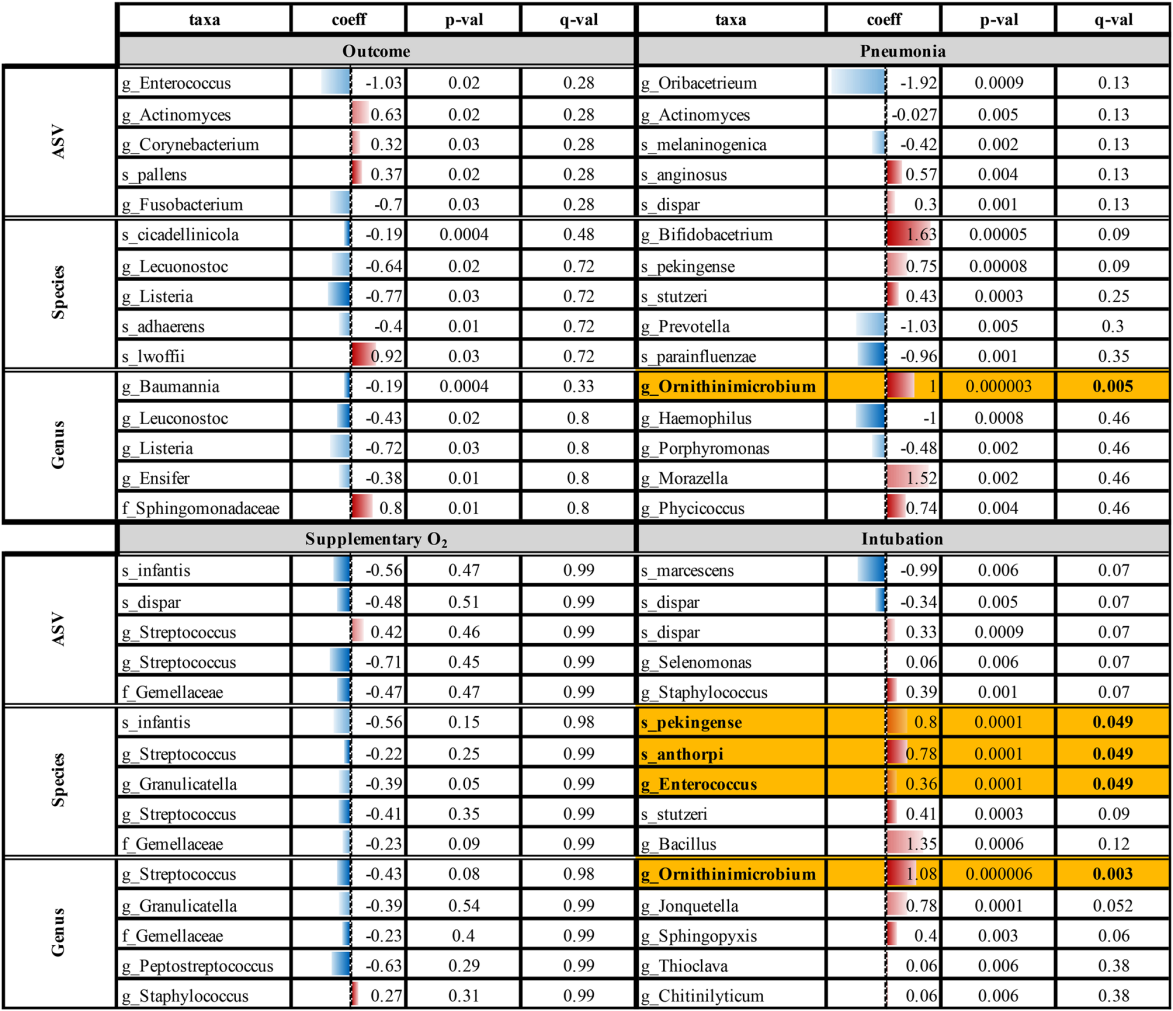
MaAsLin2 top 5 DA taxa (based on *q* values). In each panel, corresponding to a covariate, coefficient, *p* values and *q* values for each taxon are reported on columns at different taxonomic resolution, namely: ASV, Species and Genus on rows. Covariates tested: Outcome (symptomatic or asymptomatic); Pneumonia (mild, moderate or severe); Supplemental O_2_ and Intubation (yes or no). Taxa with statistically significant *q* values (< 0.05) are highlighted in bold with a golden background. Coefficient values are graphically resumed with a blue (less abundant) to red (more abundant) color scale, ranging from − 2 to 2. For greater clarity, taxa names were reported according to the last taxonomic level classified during the read preprocessing.

Among others, the genus *Ornithinimicrobium* (in particular the species *pekingense*) resulted positively differentially abundant both in patients developing severe pneumonia and in those undergoing a high flow intubation, while the species *Jonquetella anthorpi*, along with the genus *Enterococcus*, resulted overabundant when specie-level taxonomy clusterization was performed. The latter is of particular interest since its retrieval in COVID-19 hospitalized patients was already reported in the literature and demonstrated to be not nosocomial-derived^18^.

### Network analysis

To corroborate the results obtained through the DA analysis, we investigated the covariate “intubation” by performing a network inference analysis, with bacteria as nodes and edges defined by the sparCC^19^ association values, to verify whether meaningful differences would arise from a complementary analysis approach.

**Figure 3:**
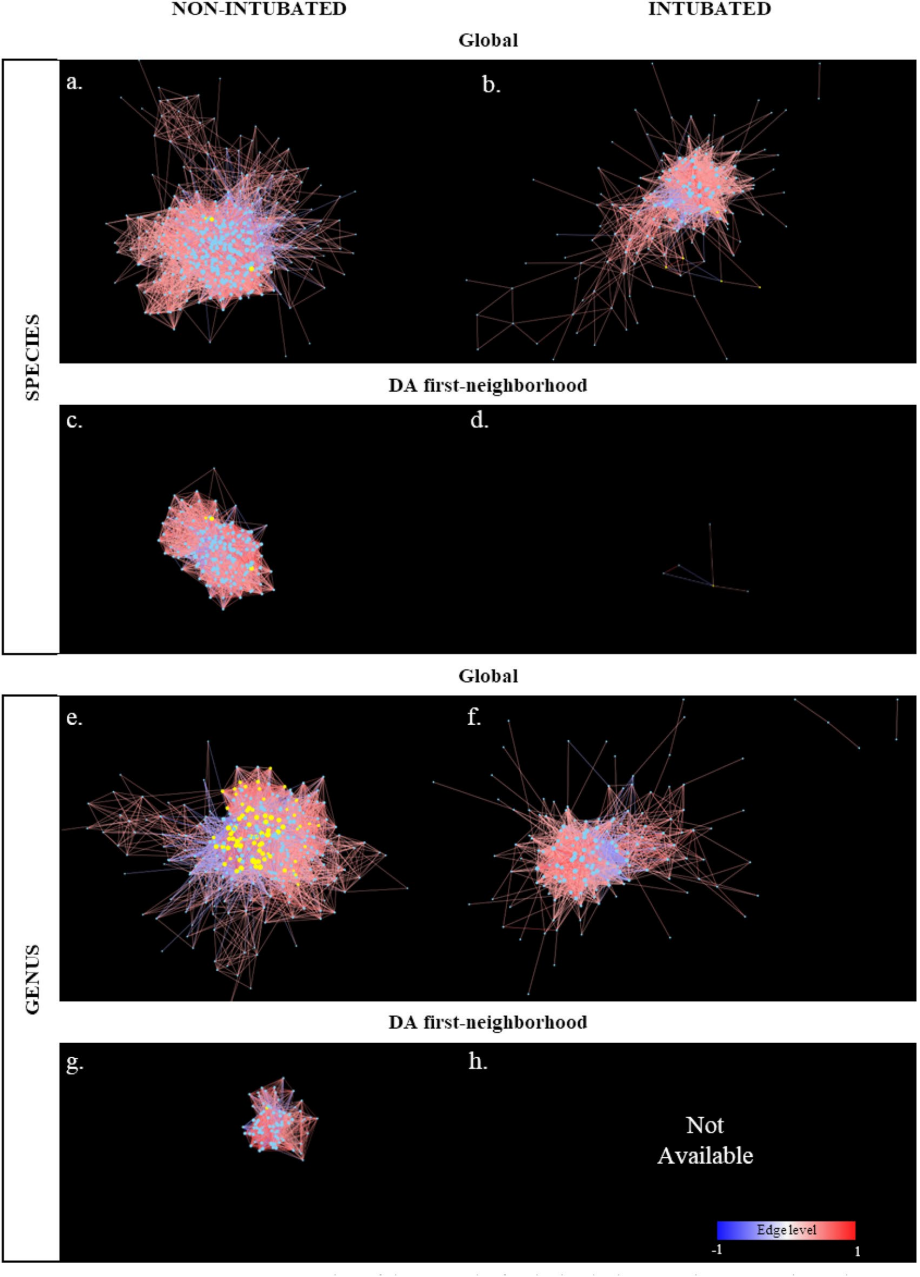
Network analysis. Cytoscape representation of the networks for the intubation covariate, at species and genus taxonomy level. Global networks refer to all the bacterial species identified in the samples while NA first neighborhood are the subnetworks connected to DA bacterial species. Nodes are the bacterial taxa, highlighted in yellow when DA; the size of the node indicates its degree. Edges are defined by the sparCC association values, with color representing their intensity, from -1 to 1, in a heat blue-red scale.

More specifically, as reported in Fig. 3a,b and e,f, two pairs of networks (intubated VS non-intubated patients) were created for the species and genus taxonomy level, respectively; edges color was set on a heat (blue to red) scale based on the association values, while nodes size recalls the degree of the node. Then, for all the four resulting networks, only the first neighborhoods – highlighted in yellow – of the DA features were selected (i.e., one node for genus, three nodes for species) obtaining the networks reported in Fig. 3c,d and g,h for species and genus taxonomy levels, respectively. Multiple metrics, reported in Table 4, were computed for the obtained networks.

**Table 4:** Network metrics. Analysis of the network properties via Cytoscape’s Analyzer.

	Full non-intubated (FN)	Full intubated (FI)	DA first-neighb.non-intubated (DAN)	DA first-neighb. intubated (DAI)
Species				
Nodes	366	171	160	5
Edges	7849	2024	4085	5
Avg. neighbors	42.89	23.94	51	2
Clustering	0.548	0.599	0.636	0.433
Density	0.118	0.143	0.321	0.500
Heterogeneity	0.673	0.955	0.380	0.548
Centralization	0.193	0.404	0.287	0.833
Genus				
Nodes	315	154	79	NA
Edges	6409	2028	1609	NA
Avg. neighbors	40.69	27.18	40.73	NA
Clustering	0.559	0.639	0.775	NA
Density	0.130	0.184	0.522	NA
Heterogeneity	0.688	0.898	0.347	NA
Centralization	0.250	0.389	0.490	NA

NA stands for not available since the network was empty.

It is worth noting that, in this case, we were not interested in finding further bacterial species of interest; indeed, the analysis was primarily performed to verify whether the specific covariate under study implies variation in network topology and connectivity and thus, in possible dysbiosis.

Comparing the full “non-intubated patients” (FN) with the “intubated patient” network (FI) at the species level, the latter results to be less populated and connected, suggesting a decrease in interactions and thus in possible regulations. However, the two networks are similar in terms of clustering coefficient and density, which highlights the presence of poorly connected nodes.

When reducing FN to its DA first-neighborhood network (DAN), nodes and edges are halves, but the average neighbors, the density, and the clustering coefficient increase. The slight increase in clustering and density, as well as the halves in heterogeneity and the increase in centralization, are in accordance with the removal of isolated nodes.

The same observations can be made considering the networks at the genus level, with rising centralization in DAN with respect to FN, along with an abrupt decrease in the number of nodes and edges. Lastly, when comparing FI to the subnetwork of the DA first-neighborhood (DAI), the size of the network collapse, depriving the metrics of their meaning and highlighting that there is no more interaction between the potential biomarkers (DA nodes) and the core taxa.

Overall, despite the connection of the network is too high to infer any property considering the whole bacterial composition, interesting aspects can still be observed for the first-neighborhood networks. Indeed, comparing the non-intubated with the intubated ones, the latter have dramatically fewer connections and nodes. This is even more clear at the genus level where the DA taxa, in intubated patients, resulted in having no connection with other taxa, thus leading to an empty network when considering DA first neighborhoods. Therefore, our data suggest that a possible complex multifactorial equilibrium involving the DA species gets lost in patients presenting a deteriorating clinical picture.

## Discussion

The main objective of this study was to find significant differences in microbial taxonomic profiles that characterize the nasopharyngeal tract of SARS-CoV-2 + patients. Each sample was collected at diagnosis. Therefore, to the best of our knowledge, this is one of the first works that do not consider healthy samples as a control. The chosen design allowed us to focus on differences in prophylaxis between patients and to find a specific bacterial composition that could promote or prevent more severe symptomatology.

Alpha and beta diversity results are in line with other studies. Although all these studies use different preprocessing pipelines, are mostly carried out at the species level, involve different numbers of subjects, and adopt different analysis tools, it can be concluded that the literature confirms a weak association between the overall microbial diversity and positive or healthy individuals. Taken together diversity analysis demonstrates that, at diagnosis, there are no hints (in terms of overall bacterial composition) about the future development of the disease. This suggests that dysbiosis of the nasopharyngeal microbiota is driven by taxa not belonging to the human core microbial community.

Although diversity analysis did not show any relevant result, the abundance of individual taxonomies resulted to be significantly different among groups of patients that undergo different treatments.

In Zhang et al.^20^, the genus *Ornithinimicrobium* was detected among the dominant bacteria in aerosols from COVID-19 patients. In addition, this genus was found to be differentially abundant at the earliest time points between control and infants that will develop lower respiratory tract infections^21^, thus reinforcing the idea that it may be a potential biomarker. *Jonquetella anthropi* has been associated with endodontic infections and periodontal diseases^22–24^. But even more interestingly, in Pragman et al.^25^ order *Synergistales*, which contains the genus *Jonquetella*, were increased in patients affected by chronic obstructive pulmonary disease. Lastly, the genus *Enterococcus*, in particular species *faecalis*, is a pathogen that causes bloodstream infection (BSI) in critically ill patients with COVID-19 in the intensive care unit^26^. Moreover, DeVoe et al.^18^ demonstrated that nosocomial transmission did not explain the increased rate of BSI due to *Enterococcus*. Also, the gut microbiome of COVID-19 patients shows enrichment of potential pathogens, particularly *Enterococcus*^2^. In nasopharyngeal microbiota, this pathogen is found differentially abundant between COVID-19-positive and -negative patients^9,27^. Since this pathogen is found in patients who undergo intubation, and a swab is performed at diagnosis, it can be suggested that it is a reliable biomarker to predict future disease progression.

It is worth noting that, in addition to the variability related to the sampling and the bioinformatic pipeline adopted for the analyses, specific taxa derived through the DA analysis could be affected by the regionality of the study. Indeed, as reported in^28^, the respiratory microbiome has geographic and climatic characteristics. This is reflected by the absence of a consensus in the DA species discovered in similar studies, conducted on patients from different countries, such as *Nisseria spp.* in Russia^28^, *Streptococcus spp.* in China^29^, *Chromobacter* and *Bacillus spp.* in India^30^.

Interestingly, the comparison between symptomatic and asymptomatic subjects does not find significant differences in terms of individual taxa. This phenomenon reinforces the idea that the DA taxa found are a signature microbiota of the upper respiratory tract, with biological interactions between these taxa and the others driving the dysbiosis.

Taken together these results show that few DA taxa could drive dysbiosis among symptomatic patients toward a severe outcome. This claim is also reinforced by the network inference analysis performed, which revealed that several complex interactions protect the patients from intubation.

Looking at the global properties of the networks, the full “intubated” one shows a certain propensity to clustering. Controversy, this observation is not informative for the identification of putative biomarkers, since it would imply that the hubs should be related to the DA taxa, while the latter result being weekly connected with the rest of the network in intubated patients.

This, on one side, suggests that the variation in the connectivity is by itself weekly prognostic for disease worsening. On the contrary, the isolation of DA nodes that arise from our analysis indicates that the DA taxa overabundance is a marker of possible disease decline. In other terms, when intubation occurs, the DA bacteria do not interact with the core of the taxa, suggesting that dysbiosis underlying SARS-CoV-2 infection allows the proliferation of those bacteria, in turn leading to prognosis worsening.

However, although the analysis demonstrates possible nodes driving the dysbiosis in patients undergoing intubation, a more in-depth analysis is needed to strengthen biological conclusions; indeed, no other network inference analysis on microbiome nasopharyngeal sequencing data is present in the literature; consequently, a complete benchmarking of network inference methods is needed to verify which is the best tool and pipeline to maximize the analysis reliability.

## Materials and methods

### Data retrieval

#### Biological samples acquisition

This study focused on the characterization of the nasopharyngeal microbiome in subjects with Sars-Cov2 infection. For each patient, the first nasopharyngeal swabs positive for SARS-COV-2 were retrieved from our collection and used for the study. As symptomatic, patients that performed the nasopharyngeal swab before hospitalization presenting at least mild symptoms of upper respiratory tract infection were enrolled, whereas nasopharyngeal swab positive for SARS-CoV-2 from asymptomatic subjects were gathered among patients involved in the national surveillance program. Between July and November 2020, nasopharyngeal swabs were collected from 194 consecutive patients; however, the actual cohort size is limited to 192 patients since the library preparation did not work for 2 samples (i.e., plate1_A6 and plate1_G6). Patients were recruited at the Infectious Disease Clinic of Padua University Hospital. Swabs were stored at − 20 °C and then microbial genomic DNA was extracted using the Ultra Deep Microbiome Prep Kit, which allows to remove DNA from eukaryotic cells and purify prokaryotic DNA. Then, DNA samples were sent for sequencing to Polo d’Innovazione di Genomica Genetica e Biologia Società Consortile R.L, (Siena, Italy).

### Ethical approval statement

The nasopharyngeal sampling was performed within the routine surveillance program established by the Veneto region. For the present study, swabs positive for SARS-COV-2 in adult patients were retrieved from the archives of the Microbiology Unit of the University Hospital of Padova; no ethical approval was required, according to National Legislation, due to the non-interventional nature of the study. After linkage to patient records, the data were anonymized and then presented in an aggregate manner.

### Sequencing technology

The V3–V4 hypervariable region of the bacterial 16S rRNA gene was sequenced using an Illumina MiSeq V2 chemistry (2 × 250 bp) after Illumina libraries prepared following the Illumina 16S Metagenomic Sequencing Library Preparation Guide (Part #15,044,223 Rev. B)^31^ and the Nextera XT Index Kit. The resulting data are registered in NCBI as Bioproject PRJNA944646.

### Patients’ metadata

In addition to sequencing data, patients’ metadata were collected considering: (i) main outcome (symptomatic, asymptomatic), (ii) different levels of pneumonia (mild, moderate, severe—as indicated in NIH classification^32^), (iii) supplemental oxygen O_2_ needed and (iv) possible subsequent intubation. Supplemental oxygen was administered to six patients with "mild" Covid for preexisting, unrelated, non-bacterial, or viral diseases (i.e., cardiac or lung disease). Not all information is relevant to all analyses. As an example, pneumonia and supplemental oxygenation needed should be used for an analysis restricted to symptomatic cases, while to examine all subjects only age, gender, and outcomes are taken into consideration, as summarized in Table 1.

### Bioinformatic pipeline

**Figure 4:**
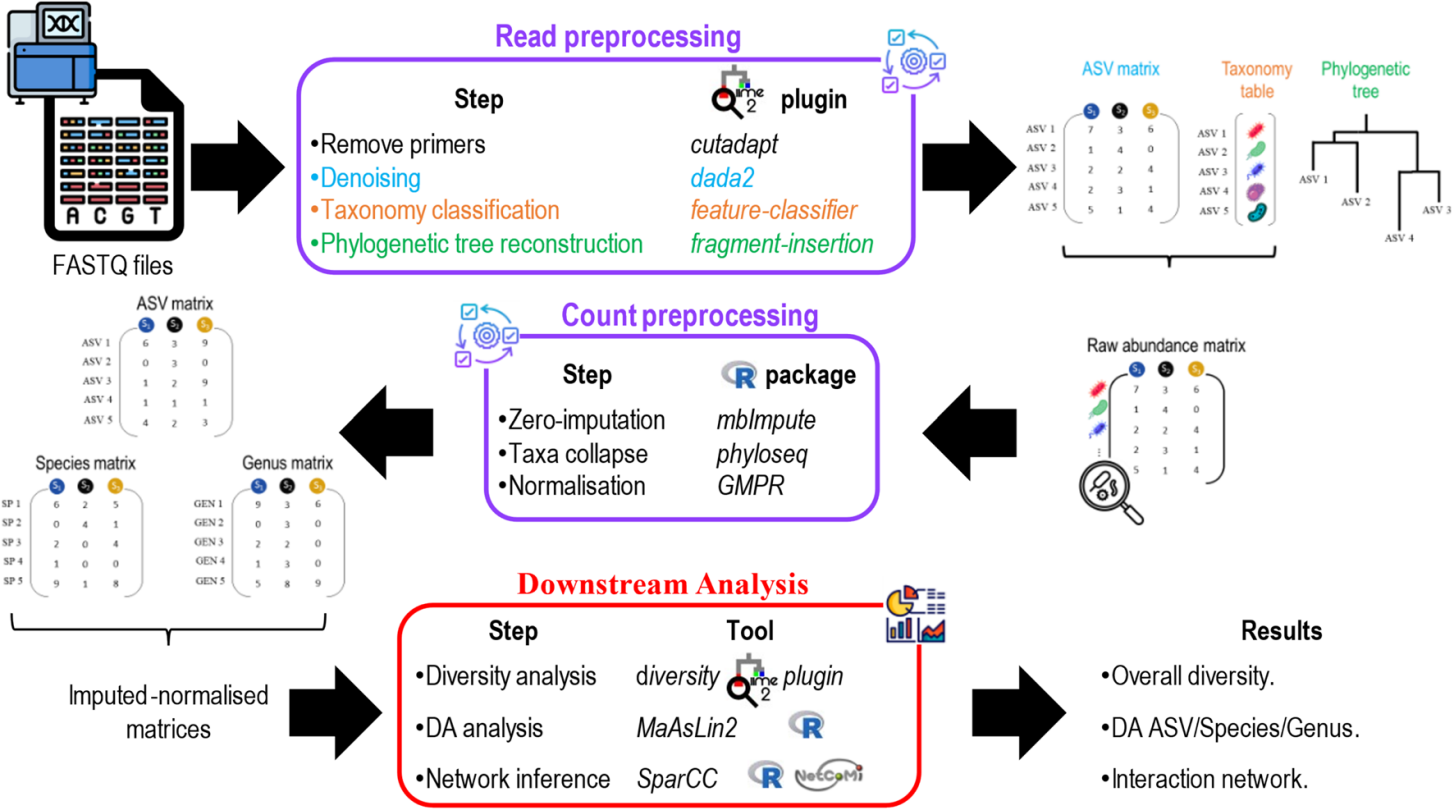
Overview of the bioinformatics pipeline.

The computational methods used to analyze the data are summarized in Fig. 4. All read preprocessing steps were performed in QIIME2 (v2021.8) software^33^, while count preprocessing in R (v4.2.0) software.

Further details about code, commands and parameters used for each pre-processing and analysis step, are available at https://gitlab.com/sysbiobig/microbiomecovid. To ensure reproducibility of results, a Docker container image containing all the software needed is available at the same link. In addition, the folder with both data and results is available on Zenodo, as reported in the Data availability section.

### Preprocessing

Sequencing data were processed for alignment and quality filtering in QIIME2 v2021.8^33^, and representative amplicon sequence variants (ASV) were obtained by the DADA2 algorithm^34^, starting from demultiplexed reads, provided by the sequencing facility. Taxonomic annotation was performed using a pre-trained naive Bayes machine-learning classifier that was trained to differentiate taxa present in the 99% Greengenes v13.8 reference database^35^ set trimmed to 250 bp of the V3-V4 hypervariable region (corresponding to the Illumina primers).

Finally, a phylogenetic tree was constructed exploiting fragment insertion approach developed by Janssen et al.^36^. Representative sequences generated during denoising were used to create a phylogenetic tree, where the sequences have been inserted into the Greengenes v13.8 99% identity reference tree backbone.

The raw abundance matrix was processed for recovering information on not detected taxa through the mbImpute R package^37^. Since the raw abundance matrix focuses purely on the unique sequence variants that were observed in each sample, groups of features that have the same taxonomic assignment in the taxonomy table were collapsed to the species and genus levels (exploiting taxa_collapse function in phyloseq R package^38^). Then, for each level of analysis (i.e., ASV, species, and genus), subject abundance profiles were normalized with the GMPR method^39^.

### Downstream analysis

The downstream analysis was focused on the differences between symptomatic and asymptomatic subjects (main outcome). Moreover, to study the relationship between the nasal microbiota and the development of a serious clinical situation, differences in symptomatic subjects were investigated considering: the level of pneumonia, the need for oxygen therapy, and the necessity of intubation. All the downstream analyses were carried out by comparing the above-mentioned groups of subjects, considering the 3 taxonomic levels: genus, species, and ASV.

#### Diversity metrics

16S rRNA-seq data were analyzed to find the characteristic microbiota traits for the clinical outcomes of interest. Statistical methods were used to evaluate significant differences in the overall microbial population of subjects’ groups, in relation to possible predictive factors of interest.

Alpha and Beta diversity analysis^16^ were performed to investigate and quantify the compositional complexity of a community within a sample and the taxonomic differences between samples, respectively.

As regards the Alpha diversity metrics, the following ones were exploited: richness, which evaluates the presence/absence of taxa; Pielou^40^, which measures how abundances are equally distributed across the different taxa; Faith^41^, which measures richness weighing taxa based on their evolutionary history, when available. Significant differences between groups were identified using the Kruskal–Wallis statistical test on each Alpha metric.

Beta diversity was investigated using different distance metrics between taxonomic profiles, such as Jaccard^42^ and Bray–Curtis^43^, together with two metrics involving the phylogenetic tree in the computation (i.e., Unweighted UniFrac and Weighted UniFrac distance^44^). Then, PCoA (Principal Coordinate Analysis) was used to perform dimensionality reduction to visualize potential group patterns considering the investigated covariates.

Diversity analysis was performed by exploiting Qiime2 Diversity and Emperor plugins.

#### Differential abundance analysis

Differential Abundance Analysis was performed using MaAsLin2^17^ R package since, as shown in^15^ this method is among the top ranking looking at the overall performance; moreover, this method is one of the few allowing to perform taxa-wise covariate adjustment and perform analysis on GMPR normalized data. The method was run with the default parameters, with covariate "sex" and "age" taxa-wise adjustment. We have run the method on the GMPR-normalized abundance matrices at the ASVs, genus, and species levels. The Wald test was chosen to test the null hypothesis of no differentially abundant taxa exploiting the Benjamini–Hochberg FDR adjustment. The commonly used threshold for the nominal α is set to 0.05.

#### Network inference analysis

SparCC^27^ was used to perform network inference analysis. Given the DA results, SparCC was run only on the species matrix for both patients that undergo or did not undergo intubation. The R package Net-Comi^45^ was exploited to infer and analyze both interaction networks in a single computational workflow. SparCC was run with default parameters, where the threshold for edge detection was set as 0.3.

The following analysis step were repeated for each investigated network: (i) Full networks were imported in Cytoscape from csv tables; (ii) duplicated and self-looping edges were removed; (iii) edge stroke color was set, in the Layout panel, from blue to red in a continuous mapping type based on the sparCC association values, i.e., from − 1 to 1; (iv) nodes size was set proportional to their degree in the Layout panel, with continuous mapping type, with a size between 5 and 50 associated to a 0 to 400 degree respectively; (v) DA nodes were selected through the Filter panel; (vi) from the selected nodes, the first neighbors were selected and used to create new subnetworks (vii) Cytoscape Analyzer was run to obtain network metrics, namely: number of nodes and edges, average number of neighbors per node, average clustering coefficient (the mean of local clustering, hence a measure of the degree to which neighbors of a node in a graph tend to link together^46^), Density (representing how densely the network is populated with edges, with a density of 0 when the network contains no edges and solely isolated nodes or 1 for fully connected networks^47^), Heterogeneity (coefficient of variation of the connectivity distribution, reflecting the tendency of a network to contain hub nodes) and Centralization (closer to 1 for networks resembling a star topology while 0 for sparse ones^48^).

## Data Availability

All the code written and used during the current study is available in the GitLab repository https://gitlab.com/sysbiobig/microbiomecovid. Anonymized data, subjects’ metadata, all files obtained through Qiime2 and R scripts, and the Cytoscape networks are available in the Zenodo repository https://doi.org/10.5281/zenodo.7713313. The sequencing reads generated during the current study are also available via the NIH Sequence Read Archive (SRA) via Bioproject PRJNA944646.
